# Disruption of Proteoglycan 4 (PRG4)-CD44 Signaling Modulates Chronic Synovitis in Conditionally Inactivated Mice

**DOI:** 10.21203/rs.3.rs-7659196/v1

**Published:** 2025-10-17

**Authors:** Khaled Elsaid, Ling Zhang, Thomas Zhao, Ava Marks, Derek Jenkins, Gregory Jay

**Affiliations:** Chapman University; Rhode Island Hospital; Boston University; Brown University; Brown University; Rhode Island Hospital

**Keywords:** Proteoglycan-4, CD44, Synovitis, Xanthine Oxidase, HIF-1a, Osteoarthritis

## Abstract

**Background:**

Proteoglycan-4 (PRG4) is a mucinous glycoprotein secreted by synovial fibroblasts and superficial zone chondrocytes. PRG4 inhibits synovial macrophage (SM) activation *via* xanthine oxidase (XO) and hypoxia inducible factor alpha (HIF-1α) suppression. We aimed to evaluate the contribution of PRG4-CD44 interaction to synovial homeostasis and investigate PRG4’s signaling dysfunction in synovial tissues from patients with osteoarthritis (OA). We hypothesized that CD44 mediates synovitis due to PRG4 loss and that PRG4 signaling dysfunction is associated with high-grade synovitis in OA.

**Methods:**

*Prg4*^*FrtloxP/FrtloxP*^ are transgenic mice wherein tamoxifen (TAM) inactivates the *Frt* allele and creates a knockout state (*Prg4*^*FrtKO/FrtKO*^*)*. TAM (*Prg4*^*Off*^) or corn oil (*Prg4*^*On*^) administration occurred in 4 weeks-old animals (4–6 animals with 2–3 males per group). We crossed this mouse with *Cd44*^−/−^ mice to generate *Cd44*^+/+^ & *Prg4*^*On*^, *Cd44*^+/+^ & *Prg4*^*Off*^, *Cd44*^−/−^ & *Prg4*^*On*^, and *Cd44*^−/−^ & *Prg4*^*Off*^. XO and HIF-1α immunostaining was conducted. Isolated SMs were activated using LPS + IFNγ and SM glycolytic activation was measured by proton efflux rate (PER). HIF-1α levels were measured by ELISA. Synovial tissues were collected from the medial and lateral joint compartments of OA patients undergoing knee arthroplasty (n = 9; 7 females and 2 males). Specimens were classified by Krenn’s synovitis score as low-grade (Score: 2–4) or high-grade (score: 5–9) synovitis. Isolated CD14 + cells were stimulated with LPS ± febuxostat, and glycolytic activation was measured by PER. Immunohistochemistry (IHC) included PRG4, CD44, XO and HIF-1α.

**Results:**

CD44 deficiency reduced XO and HIF-1α staining in addition to synovial pathology following *Prg4* inactivation (*p* < *0.05*). SMs from *Cd44*^−/−^ & *Prg4*^*Off*^ mice were less activated than *Cd44*^+/+^ *& Prg4*^*Off*^ mice (*p* < *0.001*) and had lower HIF-1α levels (*p* < *0.0001*). High-grade synovitis tissues displayed less PRG4 and greater CD44, XO and HIF-1α (*p* < *0.001*) IHC staining compared to low-grade and normal tissues. Febuxostat reduced CD14 + cell activation from medial (*p* < *0.0001*) and lateral (*p* < *0.05*) joint compartments.

**Conclusions:**

CD44 loss abrogated chronic synovitis observed following PRG4 loss. Dysfunction in PRG4 signaling, demonstrated by lower tissue levels of PRG4 along with higher CD44, XO and HIF-1α, was associated with high-grade synovitis. Targeting the downstream events of PRG4 loss is potentially therapeutic in OA synovitis.

## Background

Osteoarthritis (OA) is a chronic degenerative joint disease affecting the whole joint. While irreversible cartilage degeneration is a hallmark of OA, bone remodeling and chronic synovitis are also common features of OA [1–3]. The contribution of synovitis to the overall etiology and progression of OA has undergone a paradigm-shift, from it being a byproduct of the OA disease process to being an effector mechanism in OA pathogenesis and progression [3]. Both magnetic-resonance imaging (MRI)-detected [4] and histological evidence of synovitis [5] were associated with worsening of radiographic OA. Furthermore, in individuals with no established OA diagnosis, synovitis was associated with an accelerated OA phenotype [6]. In addition to its role in mediating structural changes in OA, synovitis was also shown to contribute to pain in OA [7]. However, clinical studies of anti-inflammatory biologics failed to show a short-term analgesic benefit in OA, and there is a lack of clinical studies examining whether controlling synovitis has a long-term disease-modifying effect [8].

The synovium is a thin, soft tissue that is comprised of a surface layer, the intima and an underlying subintima [9, 10]. The intima of normal synovium is 1–3 cell layers thick, with two cell types: fibroblast-like synoviocytes (FLS) and macrophages [9, 10]. In response to chronic inflammatory signals, the synovial lining layer becomes hypertrophic with proliferation of FLS and recruitment of pro-inflammatory monocytes that differentiate into activated macrophages [11]. The sublining layer can also be enriched in macrophages, T-cells, and to a lesser degree mast cells and B cells [11]. Triggers of synovitis are not entirely appreciated and may include multiple mechanisms [11]. One prevailing theory of synovitis is the innate immune activation of synovial macrophages (SMs) by damage-associated molecular patterns (DAMPs), generated from cartilage turnover [12, 13]. Resident SMs play an important role in the initial response to these DAMPs, where they secrete pro-inflammatory cytokines and chemokines to induce the proliferation of FLS and secretion of matrix degrading enzymes and infiltration and subsequent differentiation of pro-inflammatory monocytes, respectively [3]. The crucial role of macrophages in driving synovitis and OA disease progression is further supported by the association of CD14 + proinflammatory monocyte/macrophage accumulation in the synovium and OA disease severity in humans [14–16].

Proteoglycan-4 (PRG4) is a mucinous glycoprotein, secreted by synovial fibroblasts and superficial zone chondrocytes with a multifaceted role in the joint including anti-adhesion, boundary lubrication and inhibition of synovial overgrowth in response to mitogenic signals [17–22]. Genetic mouse models of Prg4 loss revealed significant and progressive synovial pathology, highlighted by synovial hyperplasia, accumulation of pro-inflammatory SMs and upregulation of innate immune signaling networks [22–24]. Biologically, PRG4 was shown to inhibit SM activation, mediated by CD44 receptor binding and inhibition of glycolysis *via* suppressing xanthine oxidase (XO) expression *in vitro* [24, 25]. Reactive oxygen species (ROS), generated by XO, play an effector role in SM activation via stabilization of hypoxia-inducible factor 1 alpha (HIF-1α) and inflammasome activation [24, 26, 27]. The significance of PRG4 as an innate immune regulator in the joint is further supported by its binding to TLR2 and TLR4, and its ability to inhibit TLR activation by OA synovial fluids [28, 29].

In this study, we aimed to dissect the biological significance of PRG4-CD44 interaction in relation to synovial homeostasis and SM activation. We assessed downstream changes in XO expression and HIF-1α accumulation as a result of PRG4-CD44 disruption. We also investigated whether PRG4 signaling dysregulation was associated with synovitis in patients with OA. We hypothesized that A) CD44 genetic ablation protected against development of synovitis *in vivo* following *Prg4* inactivation, and B) dysregulation of Prg4 signaling was associated with high-grade synovitis. Our approach included generating CD44 competent or null mice in otherwise *Prg4* conditionally inactivated mice [24], which are born PRG4 competent and *Prg4* is inactivated by tamoxifen administration. We assessed the extent of synovial pathology [30] in these new mice and performed immunostaining for XO, HIF-1α, CX3CR1, a marker of homeostatic monocytes/macrophages [31] and CCR2, a marker of recruited pro-inflammatory monocytes/macrophages that effectuate synovitis and cartilage destruction in OA [32]. In addition, we stained for TREM2, a marker of homeostatic SMs and claudin-5, a tight junction marker, identified in synovial lining, and reportedly expressed by barrier SMs [33]. We isolated SMs from *Prg4*^*FrtloxP/FrtloxP*^ mice that were either CD44 sufficient or null, and studied their activation status using glycolytic shift assay and supplemented our studies with ROS quantification and HIF-1α cellular level measurements. We also studied synovial tissues isolated from medial and lateral joint compartments of OA patients undergoing knee arthroplasty. We used Krenn’s synovitis score (KSS) [34] to assess the extent of synovitis in these tissues and classified specimens into low-grade or high-grade synovitis. We performed PRG4, CD44, XO and HIF-1α immunostaining and studied the dysregulation of PRG4 signaling in these specimens. Finally, we isolated CD14 + pro-inflammatory monocytes and studied whether XO inhibition protected against their pro-inflammatory activation.

## Methods

### Generation of CD44 null or CD44 competent and Prg4 conditionally inactivated mice

*Prg4*^*FrtloxP/FrtloxP*^ are transgenic mice developed by Matthew Warman MD, where the *Prg4*^*Frt*^ allele normally expresses the PRG4 protein and was designed to flank the first two exons of PRG4 with a flippase recognition target and “LOXP” sites [24]. Inducing the flippase activity with tamoxifen (TAM) inactivates the *Frt* allele and thus creates a knockout state (*Prg4*^*FrtKO/FrtKO*^*)*. TAM (0.1mg/gram) (*Prg4*^*Off*^) or corn oil (Veh) (100μl) (*Prg4*^*On*^) administration occurred in 4 weeks-old animals for 10 days and histological analyses and synovial tissue collection for SM isolations were performed 6 weeks later. We crossed our *Prg4* conditionally inactivated mice with *Cd44*^−/−^ mice (strain # 5085; Jax). Both mice share the same genetic background. *Cd44*^−/−^ & *Prg4*^*FrtloxP/FrtloxP*^ and *Cd44*^+/+^ & *Prg4*^*FrtloxP/FrtloxP*^ animals were genotyped as described by us [24] and according to vendor’s recommendation (*Cd44*). The breeding of the new mouse model was approved by the IACUC of Rhode Island Hospital (Reference #500225).

### Histological Analyses & complete blood counts

Knee joints were harvested at 6 weeks following completion of TAM or Veh administrations, decalcified, paraffin-embedded and sectioned. Histological sections (5μm) were selected to include both meniscal horns as landmarks. Immunoprobing included PRG4 (Mab S6.79 provided by Dr. Tom Schmidt) [35], CD44 (ab189524; recombinant anti-CD44 rabbit antibody), CX3CR1 (ab308613; recombinant anti-CX3CR1 rabbit antibody), CCR2 (ab273050; recombinant anti-CCR2 rabbit antibody), TREM2 (ab305103; recombinant anti-TREM2 rabbit antibody), Claudin-5 (ab131259; recombinant anti-claudin 5 rabbit antibody), XO (ab109235; recombinant anti-XO rabbit antibody) and HIF-1α (ab179483; recombinant anti-HIF-1 rabbit antibody) (Abcam) (1:100 dilutions for all antibodies) and incubated overnight at 4°C. Following washing with PBS, sections were incubated with Cy3 goat anti-mouse IgG (PRG4) or Cy3 goat anti-rabbit IgG (CD44, CX3CR1, CCR2, TREM2, claudin-5, XO and HIF-1) at 1:200 dilution for 1h at room temperature. Sections were washed with PBS and mounted in Vectashield mounting medium with DAPI (Vector Laboratories Inc.). Slides were imaged using fluorescence microscopy (Nikon, ECLIPSE E800) and quantified using Image J. Sections were also stained with hematoxylin and eosin (H&E) and synovial pathology was assessed by two blinded investigators (authors KE and GJ) using a 0–3 score, where 0 = normal synovium, 1–2 cell layers thick and 3 = severe extensive hypertrophy > 5 cell layers and/or infiltration of synovium greater than 50% of surface [30]. At the time of joint harvest, blood was collected from the 4 genotypes by cardiac puncture, and the numbers of white blood cells (WBCs), lymphocytes, monocytes and neutrophils were estimated using a hemocytometer.

### Isolation of SMs and glycolytic activation studies

Synovial tissues from 2–3 animals were pooled together and subjected to SM isolation and surface marker characterization [24]. Glycolytic activation of cultured SMs (50,000 cells per well) was monitored in real-time using a Seahorse Analyzer (Seahorse XF HS Mini Analyzer; Agilent Technologies). Proinflammatory activation was performed using LPS (100 ng/mL) and IFNγ (20 ng/mL) followed by OCR (Oxygen Consumption Rate) and ECAR (Extracellular Acidification Rate) measurements over 60 minutes. Proton Efflux Rate (PER) was calculated, and mean PER was compared across experimental groups. Proton Efflux Rate is an indicator of macrophage immune activation, and a higher PER is indicative of stronger pro-inflammatory activation [36].

### ROS and HIF-1α quantifications in Cultured SMs

Cultured SMs (50,000 cells per well) were activated using LPS (100ng/ml) and IFNγ (20ng/ml) and ROS levels were quantified at 24h using the DCFDA/H2DCFDA kit (Abcam). SMs were also collected following similar treatments and cellular HIF-1α levels were determined by ELISA (Abcam) and normalized to total protein levels.

### Synovial tissue collection from OA patients undergoing arthroplasty

A total of 9 OA patients (7 females and 2 males) were enrolled in this observational study. The median age for participants was 74 years with a range of 64 to 85. A total of 7 patients had knee arthroplasty in their right knee. Varus anomaly was present in 7 patients and valgus anomaly was present in 2 patients. Synovial tissue specimens were collected from the medial and lateral knee joint compartments of the operated knee, and the surgeon noted for each participant, the compartment where the synovial tissue appeared to be more grossly inflamed. Recovery and processing of human tissue for research purposes were authorized by the Lifespan Institutional Review Board (Human subjects Reference #412420), and written informed consent was obtained from participating donors. Two synovial tissue specimens from normal subjects were obtained from the National Disease Research Interchange (NDRI) and included as controls.

### Isolation of CD14 + monocytes/macrophages from synovial tissues and activation by LPS

Synovial tissues were cut into small pieces and incubated in 10 ml of DMEM containing 1 mg/ml of collagenase type 4 (220 U/mg; Worthington Biochemical Corp) for 3h at 37°C. CD14 Dynabeads (ThermoFisher Scientific) were used to isolate CD14 + cells by magnetic separation using a DynaMag-15 magnet (ThermoFisher Scientific). Glycolytic activation (50,000 cells per well) was performed by LPS (100 ng/ml), and PER was calculated as described above. Pharmacological treatments included febuxostat, a specific XO inhibitor, (25μM) (Cayman Chemicals) and N-acetylcysteine (NAC), a pan-ROS scavenger (5μM) (Cayman Chemicals).

### Assessment of synovitis and immunostaining of PRG4 signaling axis

H&E-stained synovial tissue sections were scored for synovitis by two blinded investigators (KE and GJ), using KSS [34]. KSS is a semi-quantitative scale with three components; synovial lining enlargement (score: 0–3), density of resident cells (score: 0–3) and inflammatory infiltrate (score: 0–3). Sum scores of 0 or 1 is classified as no synovitis while sum scores of 2–4 is classified as low-grade synovitis and sum scores 5–9 is classified as high-grade synovitis. Immunostainings included PRG4, CD44, XO and HIF-1α using the same antibodies and procedure as described above. Integrated fluorescence intensities of regions of interest in each specimen were normalized to corresponding mean intensities in normal synovial tissue specimens.

### Statistical Analyses

A mixed effects generalized linear model (GLM) with a random effect for participants was used to model PER across different treatments within a joint compartment. Intraclass correlation was calculated to assess the variance explained by the participant. Mean PER values were calculated and compared across different treatments by analysis of variance (ANOVA) for both mouse and human studies. Concordance between surgeon’s subjective assessment of synovitis and CD14 + cell stimulation or KSS values was calculated using proportions. Two group comparisons were performed using Student’s *t*-test. A *p* value of 0.05 was considered statistically significant.

## Results

### A combination of CD44 competent & Prg4 conditional inactivation drives the appearance of CCR2 Macs and is Associated with Decrease in TREM2 and Claudin-5

**Figure 1:**
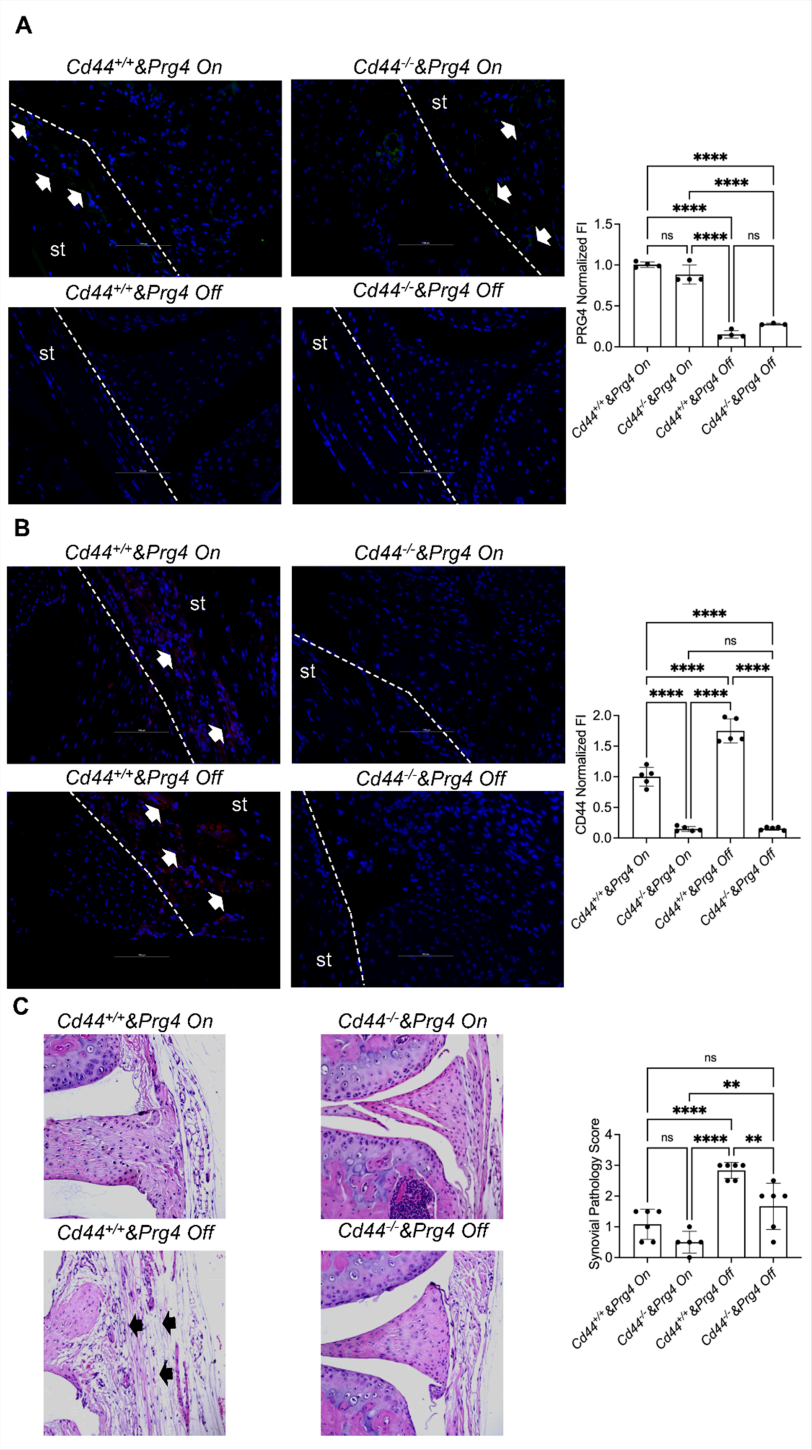
Synovial tissue (st) pathology in proteoglycan-4 (*Prg4*) conditionally inactivated mice is modulated by CD44. *Prg4* conditionally inactivated mice were crossed with *Cd44*^−/−^ mice to generate *Cd44*^−/−^ & *Prg4*^*On/Off*^ and *Cd44*^+/+^ & *Prg4*^*On/Off*^ mice. *Prg4* inactivation (*Prg4*^*Off*^) was performed using intraperitoneal tamoxifen (0.1 mg/gram) daily for 10 days starting at 4 weeks or vehicle corn oil (100ml) (*Prg4*^*On*^) and histological analyses were performed 6 weeks later. Experimental groups included 4–6 animals with each group including 2–3 males and 2–3 females. ns: non-significant; **p<0.01; ***p<0.001; ****p<0.0001. st: synovial tissue. **A.** PRG4 immunostaining was negative in the synovium of *Prg4*^*Off*^ animals. Arrows point to positive PRG4 staining **B.** CD44 immunostaining was negative in the synovium of *Cd44*^−/−^ animals. Arrows point to positive CD44 staining. **C.** *Prg4* inactivation in *Cd44*^+/+^ animals increased synovial pathology to a greater extent than *Prg4* inactivation in *Cd44*^−/−^ animals. Arrows point to synovial thickening and immune cell infiltration in the *Cd44*^+/+^ & *Prg4*^*Off*^synovium. Dashed lines outline st contour and limits.

We validated our 4 murine genotypes by Prg4 (Fig. 1A) and CD44 (Fig. 1B) immunostaining. Following TAM administration (*Cd44*^+/+^ *& Prg4*^*Off*^ and *Cd44*^−/−^ *& Prg4*^*Off*^), we observed diminution of Prg4 staining (Fig. 1A) with more than 80% reduction in integrated staining intensity compared to corn oil administered *Cd44*^+/+^ *& Prg4*^*On*^ and *Cd44*^−/−^ *& Prg4*^*On*^ synovial tissues (*p* < *0.0001* for all comparisons). Similarly, CD44 staining was absent in *Cd44*^−/−^ *& Prg4*^*On*^ and *Cd44*^−/−^ *& Prg4*^*Off*^ synovial tissues compared to *Cd44*^+/+^ *& Prg4*^*On*^ and *Cd44*^+/+^ *& Prg4*^*Off*^ synovial tissues with ~ 90% reduction in integrated intensity (*p* < *0.0001* for both comparisons) (Fig. 1B). Mean CD44 staining intensity in *Cd44*^+/+^ *& Prg4*^*Off*^ tissues was higher than *Cd44*^+/+^ *& Prg4*^*On*^ tissues (~ 75% higher) (*p* < *0.0001*) (Fig. 1B). This is consistent with our prior observations [24] and indicates that PRG4 loss upregulated CD44 in synovial tissues. Loss of CD44 expression protected against synovial pathological changes that typically occurred following *Prg4* inactivation (Fig. 1C; *p* < *0.01*). Representative images show enhanced infiltration of immune cells in synovial tissues of *Cd44*^+/+^ *& Prg4*^*Off*^ animals (indicated by arrows) compared to *Cd44*^−/−^ *& Prg4*^*Off*^. However, this protection was not complete as the mean synovial pathology score in *Cd44*^−/−^ *& Prg4*^*Off*^ animals remained higher than the corresponding value in *Cd44*^−/−^ *& Prg4*^*On*^ animals (*p* < *0.01*).

**Figure 2:**
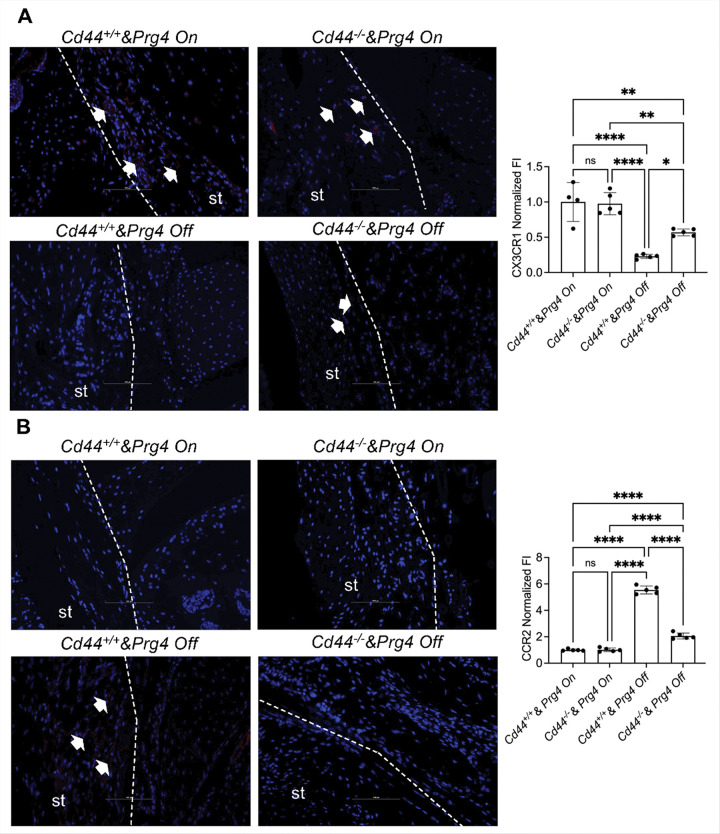
Findings in synovial tissues (st) in proteoglycan-4 (*Prg4*) conditionally inactivated mice include depletion of CX3CR1+ anti-inflammatory cells and accumulation of CCR2+ pro-inflammatory cells, and these changes were suppressed in *Cd44*^−/−^ mice. *Prg4* inactivation (*Prg4*^*Off*^) was performed using intraperitoneal tamoxifen (0.1 mg/gram) daily for 10 days starting at 4 weeks or vehicle corn oil (100ml) (*Prg4*^*On*^) and histological analyses were performed 6 weeks later. Experimental groups included 4–5 animals with each group including 2 males and 2–3 females. ns: non-significant; *p<0.05; **p<0.01; ****p<0.0001. **A.** CX3CR1 staining was attenuated to a greater extent in the *Cd44*^+/+^ & *Prg4*^*Off*^ synovium than the *Cd44*^−/−^ & *Prg4*^*Off*^ synovium. Arrows indicate positive CX3CR1 staining. **B.** CCR2 staining was enhanced to a greater extent in the *Cd44*^+/+^ & *Prg4*^*Off*^ synovium than the *Cd44*^−/−^ & *Prg4*^*Off*^ synovium. Arrows point to positive CCR2 staining. Dashed lines outline st contour and limits.

CD44 genetic ablation also corrected the imbalance between CX3CR1 + anti-inflammatory (Fig. 2A) and CCR2 + pro-inflammatory (Fig. 2B) cells in synovial tissues of *Prg4* inactivated mice. We observed ~ 250% increase in mean CX3CR1 staining in *Cd44*^−/−^ *& Prg4*^*Off*^ compared to *Cd44*^+/+^ *& Prg4*^*Off*^ synovial tissues (*p* < *0.05*). However, mean CX3CR1 staining remained lower in *Cd44*^−/−^ *& Prg4*^*Off*^ compared to *Cd44*^−/−^ *& Prg4*^*On*^ synovial tissues (*p* < *0.01*). In contrast, we observed ~ 63% reduction in mean CCR2 staining in *Cd44*^−/−^ *& Prg4*^*Off*^ compared to *Cd44*^+/+^ *& Prg4*^*Off*^ synovial tissues (*p* < *0.0001*). However, mean CCR2 staining remained higher in *Cd44*^−/−^ *& Prg4*^*Off*^ compared to *Cd44*^−/−^ *& Prg4*^*On*^ synovial tissues (*p* < *0.0001*). A similar pattern was also observed for TREM2 and claudin-5 staining, where CD44 ablation in *Prg4* inactivated mice increased TREM2 staining by ~ 67% (*p* < *0.05*; **supplementary Fig. 1A**) and claudin-5 by ~ 90% (*p* < *0.001*; **supplementary Fig. 1B**). In CD44 competent mice, *Prg4* inactivation increased circulating WBCs (**Supplementary Fig. 2A**; *p* < *0.0001*), lymphocytes (**Supplementary Fig. 2B**; *p* < *0.0001*), monocytes (**Supplementary Fig. 2C**; *p* < *0.001*) and granulocytes (**Supplementary Fig. 2D**; *p* < *0.01*). In CD44 null mice, *Prg4* inactivation didn’t significantly alter the number of circulating immune cells under study. In addition, the difference in WBCs and monocytes’ numbers between *Cd44*^+/+^ *& Prg4*^*Off*^ and *Cd44*^−/−^ *& Prg4*^*Off*^ animals reached statistical significance (*p* < *0.01* for both comparisons). Taken together, this data indicates that CD44 genetic deletion attenuated inflammation, both locally in the joint as well as systemically in otherwise PRG4 deficient mice.

### Cd44 knockout protected against XO upregulation and HIF-1α accumulation in the synovium following Prg4 inactivation

**Figure 3:**
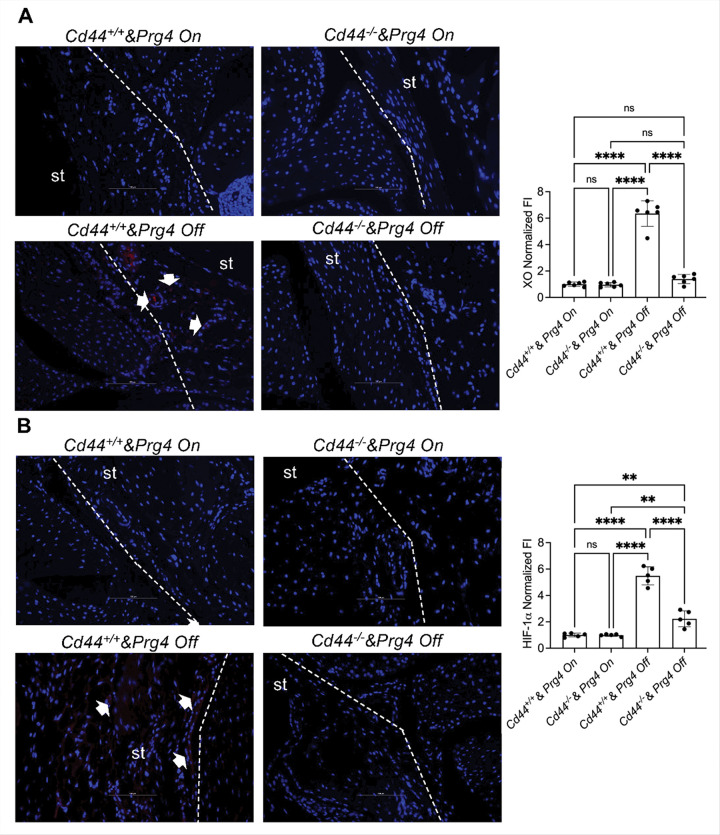
Modulation of PRG4 downstream signaling effectors; xanthine oxidase (XO) and hypoxia-inducible factor alpha (HIF-1a) in synovial tissues (st) by CD44. *Prg4* conditionally inactivated mice were crossed with *Cd44*^−/−^ mice to generate *Cd44*^−/−^ & *Prg4*^*On/Off*^ and *Cd44*^+/+^ & *Prg4*^*On/Off*^ mice. *Prg4* inactivation (*Prg4*^*Off*^) was performed using intraperitoneal tamoxifen (0.1 mg/gram) daily for 10 days starting at 4 weeks or vehicle corn oil (100ml) (*Prg4*^*On*^) and histological analyses were performed 6 weeks later. Experimental groups included 5–6 animals with each group including 2–3 males and 2–3 females. ns: non-significant; **p<0.01; ****p<0.0001. st: synovial tissue. **A.** XO staining was higher in the *Cd44*^+/+^ *& Prg4*^*Off*^ synovium compared to the *Cd44*^−/−^ & *Prg4*^*Off*^ synovium. Arrows point to positive XO staining. **B.** HIF-1a staining was higher in the *Cd44*^+/+^ *& Prg4*^*Off*^ synovium compared to the *Cd44*^−/−^ & *Prg4*^*Off*^ synovium. Arrows indicate positive HIF-1a staining. Dashed lines outline st contour and limits.

Our XO and HIF-1α staining in synovial tissues of *Prg4* inactivated & CD44 competent mice reinforce our prior findings [24] that *Prg4* inactivation induced XO (Fig. 3A; ~6-fold increase; *p* < *0.0001*) and resulted in HIF-1α accumulation (Fig. 3B; ~5-fold increase; *p* < *0.001*). CD44 knockout reduced XO induction by ~ 80% (*p* < *0.0001*) following *Prg4* inactivation and normalized XO content similar to that in *Cd44*^+/+^ *& Prg4*^*On*^ and *Cd44*^−/−^ *& Prg4*^*On*^ tissues (*p > 0.05* for both comparisons). Likewise, CD44 knockout reduced HIF-1α accumulation by ~ 60% (*p* < *0.0001*) following *Prg4* inactivation. However, mean HIF-1α staining in *Cd44*^−/−^ *& Prg4*^*Off*^ synovial tissues remained higher than in *Cd44*^+/+^ *& Prg4*^*On*^ and *Cd44*^−/−^ *& Prg4*^*On*^ (*p* < *0.01* for both comparisons).

**Figure 4:**
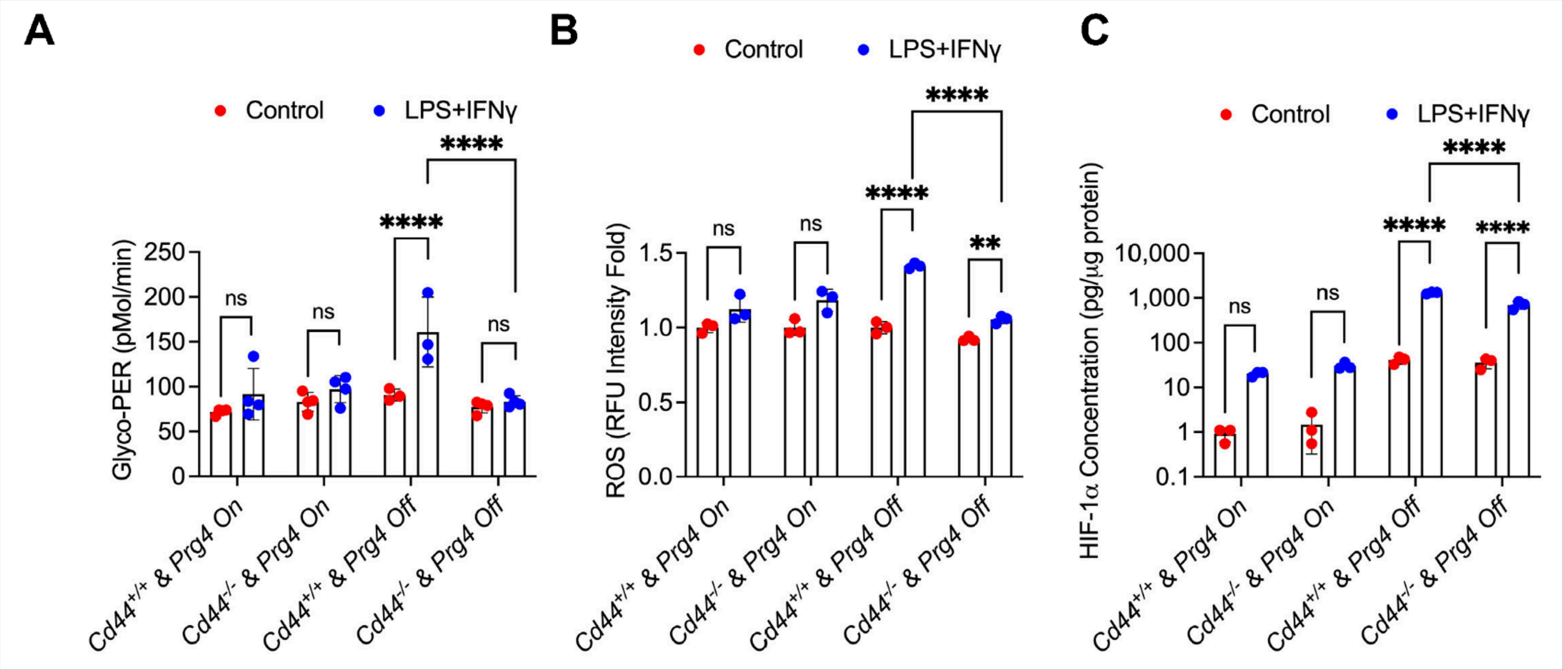
Proinflammatory activation status of synovial macrophages (SMs) and its relationship to hypoxia-inducible factor alpha (HIF-1a) levels from proteoglycan-4 (*Prg4*) conditionally inactivated and *Cd44* null mice. *Prg4* conditionally inactivated mice were crossed with *Cd44*^−/−^ mice to generate *Cd44*^−/−^ & *Prg4*^*On/Off*^ and *Cd44*^+/+^ & *Prg4*^*On/Off*^ mice. *Prg4* inactivation (*Prg4*^*Off*^) was performed using intraperitoneal tamoxifen (0.1 mg/gram) daily for 10 days starting at 4 weeks or vehicle corn oil (100ml) (*Prg4*^*On*^) and SM isolation was performed 6 weeks later. Pro-inflammatory activation was achieved by treatment with LPS (100 ng/ml) and IFNg (20 ng/ml), and glycolytic proton efflux rate (Glyco-PER) was measured. ROS was determined fluorometrically. HIF-1a levels were measured by ELISA and normalized to total protein. ns: non-significant; **p<0.01; ****p<0.0001. **A.** Pro-inflammatory activation was stronger in SMs from *Cd44*^+/+^ & *Prg4*^*Off*^ compared to *Cd44*^−/−^ & *Prg4*^*Off*^. **B.** ROS levels were higher in LPS+IFNg treated SMs from *Cd44*^+/+^ & *Prg4*^*Off*^ compared to *Cd44*^−/−^ & *Prg4*^*Off*^. **C.** HIF-1a levels were higher in LPS+IFNg treated SMs from *Cd44*^+/+^ & *Prg4*^*Off*^ compared to *Cd44*^−/−^ & *Prg4*^*Off*^.

The pro-inflammatory activation status of isolated SMs was investigated by assaying their glycolytic shift in response to LPS and IFNγ (Fig. 4A). SMs from *Cd44*^+/+^ *& Prg4*^*On*^ and *Cd44*^−/−^ *& Prg4*^*Off*^ animals were not significantly activated by LPS + IFNγ. In contrast, SMs from *Cd44*^+/+^ *& Prg4*^*Off*^ animals exhibited a robust glycolytic shift in response to LPS + IFNγ indicating their pro-inflammatory activation status. However, *Cd44*^−/−^ *& Prg4*^*Off*^ SMs failed to elicit a glycolytic shift as their mean PER values remained significantly lower (*p* < *0.0001*) than corresponding values in *Cd44*^+/+^ *& Prg4*^*Off*^ SMs. A similar trend was also observed in ROS generated in SMs in response to LPS + IFNγ (Fig. 4B). CD44 knockout suppressed ROS in *Prg4* inactivated SMs (*p* < *0.0001* between *Cd44*^−/−^ *& Prg4*^*Off*^ and *Cd44*^+/+^ *& Prg4*^*Off*^ SMs). We also measured cellular HIF-1α levels and discovered that LPS + IFNγ treatment increased HIF-1α levels in *Cd44*^+/+^ *& Prg4*^*Off*^ and *Cd44*^−/−^ *& Prg4*^*Off*^ SMs (Fig. 4C; *p* < *0.0001* for both comparisons). We also observed that HIF-1α levels were lower in *Cd44*^−/−^ *& Prg4*^*Off*^ compared to *Cd44*^+/+^ *& Prg4*^*Off*^ SMs (*p* < *0.0001*). Taken together, our data suggests that CD44 knockout protected against SM pro-inflammatory activation, mediated by lower ROS generation and HIF-1α accumulation.

### CD14 + cells from the medial joint compartment were strongly activated by LPS and febuxostat treatment reduced glycolytic shift of CD14 + cells from the medial and lateral joint compartments in OA patients

**Figure 5:**
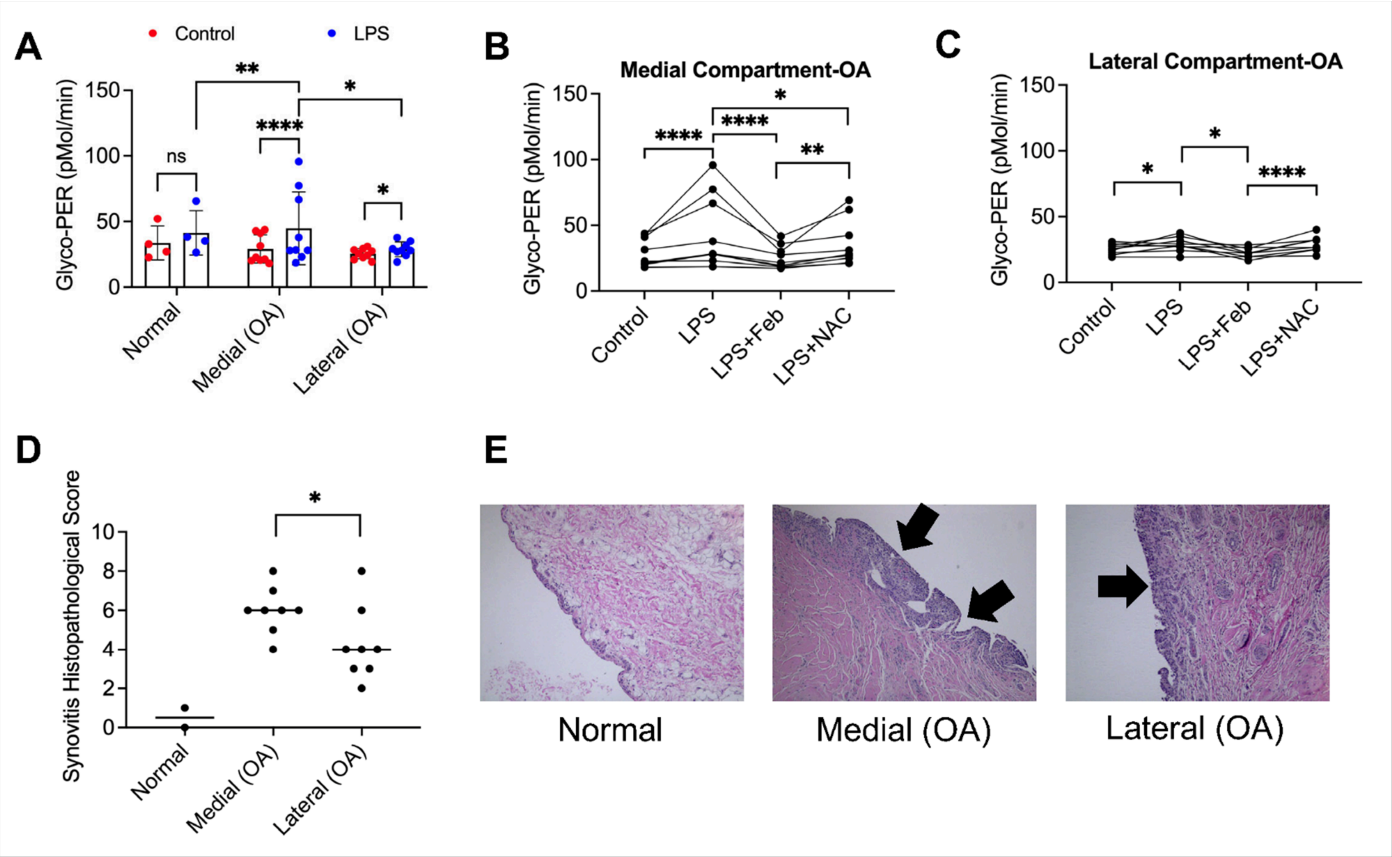
Pro-inflammatory activation status of CD14+ immune cells from synovial tissues from patients with osteoarthritis (OA) undergoing knee arthroplasty. A total of 9 patients (2 males and 7 females) underwent synovial biopsies from the medial and lateral knee compartments and CD14+ cells were liberated and studied for their pro-inflammatory activation status using glycolytic proton efflux rate (Glyco-PER) and compared to normal subjects (n=2). LPS treatment (1ng/ml) was performed and the anti-inflammatory activities of febuxostat (xanthine oxidase inhibitor) or N-acetylcysteine (NAC) (pan reactive oxygen species scavenger) were evaluated. Severity of synovitis was assessed using the Krenn histopathological scoring system. ns: non-significant; *p<0.05; **p<0.01; ****p<0.0001. **A.** CD14+ cells from the medial compartment were activated by LPS to a greater extent compared to CD14+ cells from the lateral compartment and from normal subjects. **B.** Febuxostat treatment reduced pro-inflammatory activation of CD14+ cells from the medial compartment to a greater extent compared to NAC. **C.** Febuxostat treatment reduced pro-inflammatory activation of CD14+ cells from the lateral compartment while NAC treatment had no effect. **D.** Chronic synovitis was more pronounced in synovial tissue from the medial joint compartment compared to the lateral compartment. Representative images show enhanced cell density and inflammatory cell infiltration (shown by arrows) in the medial compartment compared to the lateral joint compartment and normal synovial tissue.

We studied the glycolytic shift of CD14 + cells, isolated from the medial and lateral joint compartments of OA patients undergoing knee arthroplasty (n = 9) or normal subjects (n = 2) (Fig. 5A). There was no observable increase in mean PER values for normal subjects. CD14 + cells from lateral joint compartments were slightly activated (*p* < *0.05*), with ~ 8% increase in mean PER values with LPS treatment. CD14 + cells from medial joint compartments were strongly activated (*p* < *0.0001*), with ~ 75% increase in mean PER values with LPS treatment. Febuxostat and NAC treatments reduced proinflammatory activation of CD14 + cells from medial joint compartments (Fig. 5B), with febuxostat showing a greater capacity to reduce CD14 + activation (*p* < *0.01* for LPS + Feb vs. LPS + NAC). In CD14 + cells from lateral joint compartments, only febuxostat treatment reduced pro-inflammatory activation (Fig. 5C) (*p* < *0.05* for LPS + Feb vs. LPS). Our findings indicate that ROS derived from XO play an important role in mediating pro-inflammatory activation of CD14 + cells, isolated from OA synovial tissues.

### CD14 + cell stimulations were concordant with synovitis grades and high-grade synovitis tissues demonstrated higher PRG4 signaling dysfunction

Krenn synovitis scores (KSS) in Fig. 5D reveal that normal synovial tissues had scores of 0 or 1, corresponding to no synovitis. The median score of the medial specimens was 6 (range: 4–8) corresponding to high-grade synovitis. The median score of the lateral specimens was 4 (range: 2–8) corresponding to low-grade synovitis. Overall, mean KSS was higher in medial synovial tissues compared to lateral tissues (*p* < *0.05*). Representative images showing the median KSS for normal, medial and lateral synovial tissues are presented in Fig. 5E. Medial compartment tissues were more likely to show more prominent immune cell infiltrates and thickening of the synovial lining layer compared to lateral compartment specimens and these changes were absent in normal specimens. We observed a 100% concordance between magnitude of activation of CD14 + cells and KSS values in the medial vs. lateral synovial tissues from each patient, as the joint compartment with the stronger CD14 + cell activation also had a higher KSS value. We also observed a 78.78% concordance between CD14 + cell activation and the clinical evaluation by the surgeon, since in 7 out of 9 OA patients, the compartment with higher CD14 + cell activation values was judged by the surgeon to have had higher inflammation. A varus anomaly was also documented in 85.7% of patients judged to have more medial compartment gross inflammatory changes, and a valgus anomaly was found in 50% of patients with more lateral compartment inflammatory changes.

**Figure 6:**
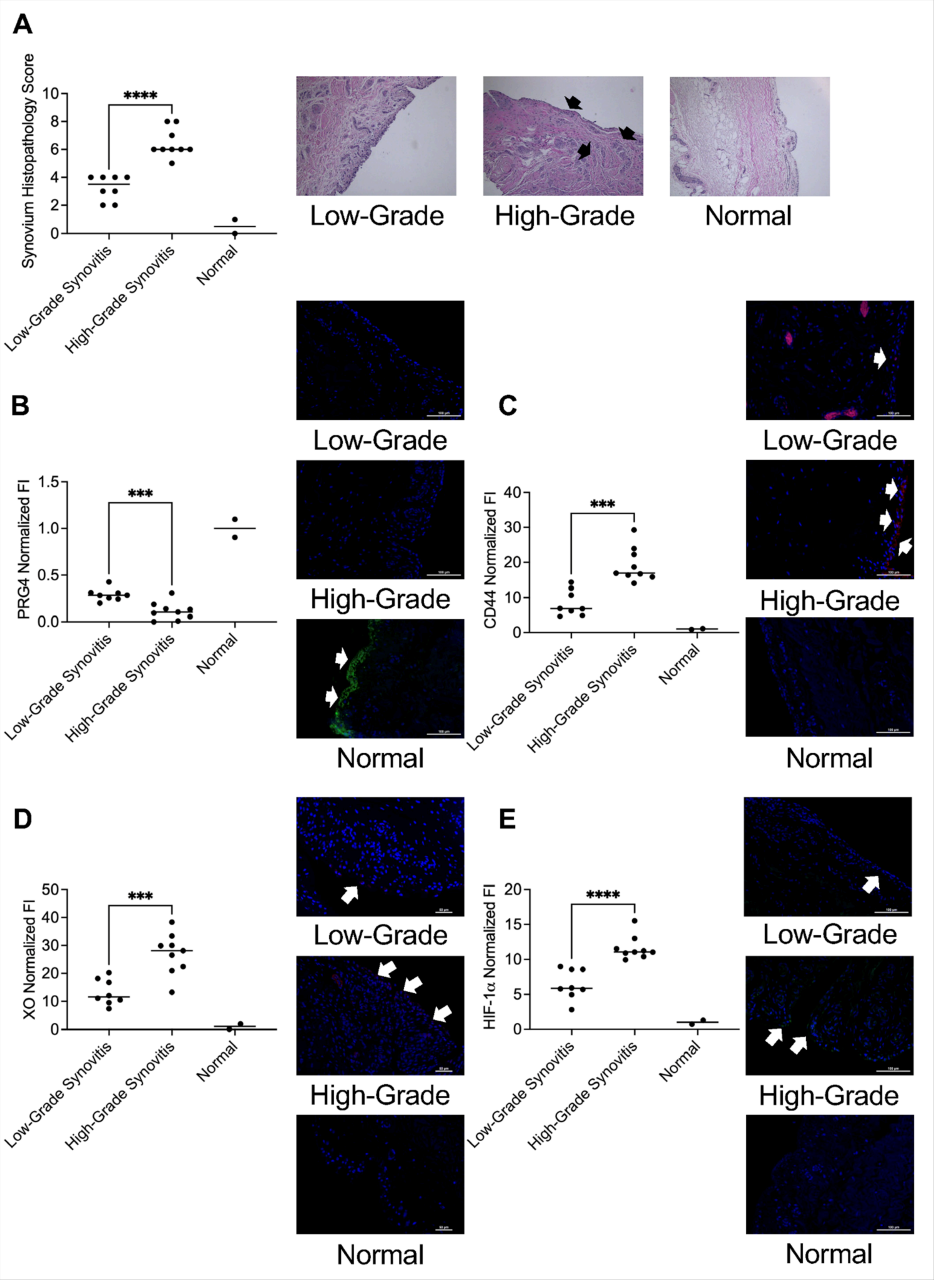
Disruption of the proteoglycan-4 (PRG4)/CD44 signaling axis is associated with higher grade synovitis in synovial tissue from medial and lateral joint compartments from patients with OA undergoing knee arthroplasty. Synovial histopathology was assessed by Krenn scoring system and biopsies were classified as high-grade (Krenn score: 5–9) or low-grade (Krenn score: 2–4). Immunostaining of PRG4, CD44, xanthine oxidase (XO) and hypoxia-inducible factor alpha (HIF-1a) was performed, and fluorescence intensities (FI) were quantified and normalized to FI of synovial biopsies from normal subjects (n=2). ***p<0.001; ****p<0.0001. **A.** High-grade synovitis tissue samples exhibited extensive lining layer hyperplasia, cell density and inflammatory cell infiltration (shown by arrows). **B.** PRG4 staining was lower in high-grade synovitis samples compared to low-grade and normal tissue. **C.** CD44 staining was higher in high-grade synovitis samples compared to low-grade and normal tissue. **D.** XO staining was higher in high-grade synovitis samples compared to low-grade and normal tissue. **E.** HIF-1astaining was higher in high-grade synovitis samples compared to low-grade and normal tissue. Arrows indicate positive staining.

We classified OA synovial tissues into low-grade synovitis (scores of 2–4) or high-grade synovitis (scores of 5–9) (n = 9 in each group) (Fig. 6A). Specimens with high-grade synovitis uniformly had higher immune cell infiltration (as shown by arrows in representative images). We studied the PRG4 signaling axis in these specimens by immunostaining and then computed the integrated intensities in our regions of interest and normalized these intensities to normal tissues. As expected, normal tissues displayed strong PRG4 staining in the lining layer (Fig. 6B). PRG4 staining decreased in both high and low-grade synovitis and PRG4 staining in high-grade synovitis specimens was lower than low-grade synovitis specimens (*p* < *0.001*). We detected CD44 staining in low and high-grade synovitis specimens but not normal tissues (Fig. 6C). Mean CD44 staining intensity was higher in high-grade synovitis compared to corresponding mean intensity in low-grade synovitis (Fig. 6C) (*p* < *0.001*). A similar pattern for XO (Fig. 6D) and HIF-1α (Fig. 6E) staining was also observed, where high-grade synovitis specimens had stronger staining for XO (*p* < *0.001*) and HIF-1α (*p* < *0.0001*) compared to low-grade synovitis specimens. Overall, high-grade synovitis specimens were more likely to have lower PRG4 content, along with higher CD44, XO and HIF-1α contents compared to low-grade synovitis specimens. Taken together, this suggests that dysfunction in PRG4 signaling is more pronounced in high-grade synovitis and may be causally related to the excess inflammation seen in these specimens.

## Discussion

In this study, we deciphered the biological impact of disrupting PRG4-CD44 interaction on synovial homeostasis and discovered that PRG4 loss upregulated CD44 in synovial tissues with an associated infiltration of CCR2 + pro-inflammatory immune cells and diminution of claudin-5 expressing barrier CX3CR1 + TREM2 + anti-inflammatory SM localization in the synovium [24, 33]. In addition to CD44 upregulation, we also observed XO and HIF-1α upregulation, both of which constitute the effector signaling pathway that triggers SM pro-inflammatory activation due to PRG4 loss [24]. In our model, CD44 was permissive for the innate immune response in synovial tissues of *Prg4* null mice (supplementary Fig. 3). CD44 served as an on/off switch where in the absence of PRG4, it is in the “on” position which resulted in inducing XO and HIF-1α, and the latter orchestrated SM pro-inflammatory activation [24, 27]. CD44 loss (in its null state) ameliorated synovial hyperplasia and overall synovial pathology seen in the *Prg4* null state and re-populated the synovium with claudin-5 expressing CX3CR1 + TREM2 + anti-inflammatory SMs. In addition, CD44 loss reduced the CCR2 + pro-inflammatory cell infiltrates, which likely contributed to amelioration of synovitis since the infiltration of Ly6C^high^ CX3CR1^low^ CCR2^high^ classical monocytes promotes joint inflammation [37]. The mechanistic link of CD44 loss and amelioration of chronic synovitis is possibly related to the role that CD44 plays in macrophage activation. The chronic synovial hyperplasia and associated inflammation seen in *Prg4* null animals was driven by pro-inflammatory macrophage accumulation since macrophage depletion protected against development of synovitis [22, 23, 30]. In SMs isolated from *Prg*4 conditionally inactivated mice, CD44, XO and HIF-1α expressions were elevated which resulted in stronger proinflammatory activation [24]. CD44 genetic ablation in these SMs protected against pro-inflammatory activation, as PER values did not significantly increase in response to TLR4 stimulation. This protection was mediated by reduced XO suppression and hence ROS generation and reduced HIF-1α accumulation. CD44 loss also protected against systemic inflammation, as defined by numbers of circulating immune cells. Of note, CD44 ablation didn’t completely protect against synovial pathology in the setting of *Prg4* inactivation. A plausible explanation for this observation is the potential role of TLR2 and TLR4 signaling in the pathogenesis of synovitis [38]. PRG4 also binds TLR2 and TLR4, and since PRG4 binds CD44 with higher affinity vis-à-vis TLR receptors [25, 28], the TLR pathway may still be activated in the mice with *Prg4* inactivation and CD44 knockout state.

CD44 is a single pass non-kinase transmembrane receptor that plays an important role in inflammation and shedding or internalization of the CD44 extracellular domain, as in the case with PRG4, induces a conformational change in its intracellular domain that activates protein phosphatase 2A (PP2A), which is anti-inflammatory *via* inhibition of NFκB nuclear translocation [21, 39]. CD44 responds to multiple stimuli in the cell microenvironment including mechanical, immune and metabolic signals. In monocytes and macrophages, CD44 is important for trafficking of inflammatory monocytes to the site of inflammation as well as pro-inflammatory activation of tissue-resident macrophages [40]. CD44 deficient murine macrophages released less IL-1β in response to TLR2 or TLR4 agonists, indicating protection against pro-inflammatory activation [40, 41]. In macrophages, CD44 ablation may prevent pro-inflammatory activation via promoting glycolysis-to-oxidative phosphorylation shift [24, 42]. CD44 upregulation transduces its intracellular signal via XO induction and PP2A inactivation, which promotes NFκB translocation and NLRP3 inflammasome activation [43]. ROS derived from XO promote NLRP3 inflammasome activation and stabilization and increase the half-life of HIF-1α [27]. In our conditionally inactivated *Prg4* and *Cd44* knockout mice, SMs failed to respond to LPS, and no glycolytic shift was observed. The CD44 loss represented the “turn-off” switch that prevented ROS generation and HIF-1α stabilization. An interesting finding in our study was the increase in CD44 expression in synovial tissues from OA patients compared to normal subjects. Interestingly, the enhancement in CD44 expression was directly related to synovitis grade, where high-grade synovitis tissues exhibited higher CD44 expression compared to tissues with low or no inflammation. This finding supports that CD44 is potentially implicated in the development of chronic synovitis, and thus a driver of OA disease. Supporting this involvement is that the expression of XO and HIF-1α trended in the same direction as CD44, where in tissues with high-grade synovitis, we observed stronger XO and HIF-1α staining, while absent in normal tissues (Supplementary Fig. 4).

PRG4 turnover dynamics is altered in inflammatory joint conditions, due to a combination of reduced expression and enhanced proteolytic degradation. PRG4 expression from synovial fibroblasts is reduced by IL-1β and increased by TGF-β, and is proteolytically degraded by elastases and cathepsins [44–46]. Protein level investigations have shown a reduction in PRG4 levels [47] and elevated friction measured *in vitro* by catabolic synovial fluid [48]. Not much is known about how PRG4 content in the synovial tissues is altered as OA progresses. Some reports conclude that PRG4 levels are high in osteoarthritic joints [49] but have not excluded that the PRG4 is partially digested or fragmented. Synovial tissue PRG4 content is arguably more biologically influential in the progression of synovitis compared to synovial fluid PRG4 content since the former is more bioavailable to interact with SMs and other innate immune cells exert its immunomodulatory role. In our study, we identified that PRG4 synovial content had an inverse relationship with synovitis grade. The reduction in PRG4 synovial content is potentially due to a reduction in its synovial expression by the inflammatory milieu in the synovium, as restoring PRG4 signaling in the synovium, *via* XO inhibition, was anti-inflammatory [24]. To test whether restoring PRG4 signaling in human OA synovium is anti-inflammatory, we isolated CD14 + cells from OA synovial tissues and stimulated these cells with a TLR4 agonist to simulate acute synovitis. Using febuxostat as a prototypical XO inhibitor, we were able to demonstrate that inhibiting the effector signaling pathway due to PRG4 loss is anti-inflammatory. The efficacy of febuxostat was more pronounced in CD14 + cells isolated from high-grade synovitis, and this correlated with the stronger XO staining observed in these specimens. These findings support a potential new role for febuxostat as an anti-inflammatory treatment for chronic synovitis in OA. This new role extends the therapeutic utility of febuxostat, which also reduces synovitis in patients with gouty arthritis [50].

In summary, we have generated a CD44 null and *Prg4* conditional knockout mouse to delineate the contribution of PRG4-CD44 interaction to synovial homeostasis and identified that CD44 loss protected against synovitis in *Prg4* conditionally inactivated mice. Furthermore, CD44 loss suppressed XO-HIF-1α signaling in SMs and restored the homeostatic equilibrium between anti-inflammatory TREM2 + CX3CR1 + SMs and pro-inflammatory CCR2 + SMs. In OA synovial tissues, a reduction in PRG4 content was associated with CD44 upregulation and induction of XO-HIF1α. CD14 + immune cells from high-grade synovitis tissues were activated by LPS and an XO inhibitor prevented this activation, indicating a potential role for XO inhibition in treatment of synovitis in OA.

### Limitations:

We examined the role of Prg4-CD44 in model mice focusing on the synovium using one end point at 6 weeks following *Prg4* inactivation. We didn’t assess long-term impact of disrupting PRG4-CD44 interaction on synovial reactivity, and we did not study the impact of synovitis modulation on cartilage health. We also focused our human tissue study on end-stage OA disease and did not assess synovial PRG4 signaling dysfunction following an acute knee injury or in early-stage OA.

## Conclusions

PRG4-CD44 signaling mediates synovial inflammation, where CD44 loss abrogates synovitis due to PRG4 dysfunction. High-grade synovitis is associated with greater PRG4-CD44 signaling dysfunction and inhibition of XO prevents pro-inflammatory activation of CD14 + cells isolated from patients with high-grade synovitis.

## Supplementary Material

Supplementary Files

This is a list of supplementary files associated with this preprint. Click to download.

• SupplementaryFigure1.tiff

• SupplementaryFigure2.tiff

• SupplementaryFig3.tif

• SupplementaryFig4.tif

## Data Availability

The data generated in this work are available from the corresponding author upon request.

## Abbreviations

CD44: Cluster of differentiation 44
DAMPs: Damage-Associated Molecular Patterns
ECAR: Extracellular Acidification Rate
ELISA: Enzyme Linked Immunosorbent Assay
FLS: Fibroblast-like synoviocytes
HIF-1a: Hypoxia inducible factor alpha
IFN-g: interferon gamma
IHC: Immunohistochemistry
IL-1b: Interleukin 1 beta
LPS: lipopolysaccharide
NFkB: Nuclear factor kappa B
OA: Osteoarthritis
OCR: Oxygen Consumption Rate
PER: Proton Efflux Rate
PRG4: Proteoglycan 4
ROS: Reactive oxygen species
SM: Synovial Macrophage
TAM: Tamoxifen
TLR: Toll-like receptor
XO: Xanthine oxidase
